# Intraoperative Cell Salvage in Oncologic Surgery: A Comprehensive Review

**DOI:** 10.3390/jcm14134786

**Published:** 2025-07-07

**Authors:** Ward H. van der Ven, Markus W. Hollmann

**Affiliations:** 1 Department of Anesthesiology, University of Amsterdam, Amsterdam UMC, Meibergdreef 9, 1105 AZ Amsterdam, The Netherlands; 2 Laboratory of Experimental Intensive Care and Anesthesiology, University of Amsterdam, Amsterdam UMC, Meibergdreef 9, 1105 AZ Amsterdam, The Netherlands

**Keywords:** autologous blood transfusion, erythrocyte transfusion, operative blood salvage, surgical oncology

## Abstract

Intraoperative cell salvage (ICS) is a blood conservation technique utilized in major surgery, yet its application in oncologic procedures remains debated. Concerns persist about the theoretical risk of metastasis through reinfusion of tumor cells, despite the established disadvantages of allogeneic blood transfusion (ABT), such as transfusion-related reactions and immunosuppression. In this review, we discuss the historical development of ICS, the technical processes of ICS including leukocyte depletion filtration and irradiation, and experimental and clinical data regarding its safety and efficacy. In vitro studies suggest that tumor cells undergo significant structural alterations during ICS processing, and additional filtration further reduces cell load, although complete removal is not always achieved. Observational studies of predominantly moderate quality, aggregated in multiple systematic reviews, consistently report no increased recurrence rates or reduced disease-free and overall survival in patients receiving ICS. Accordingly, national and international guidelines endorse the use of ICS during oncologic surgery. Although high-quality data—preferably from randomized controlled trials—are lacking, and certainty of available evidence from observational studies is low, ICS appears to be effective and safe. The broader adoption of its use during oncologic surgery may be warranted to minimize reliance on ABT and its associated risks.

## 1. Introduction

Intraoperative cell salvage (ICS) is a method of collecting and reinfusing autologous blood lost during surgery. It has become standard of care for various surgical disciplines, including cardiovascular and orthopedic surgery, to reduce allogeneic blood transfusions (ABT). Despite hereby avoiding disadvantages of ABT such as hemolytic transfusion reactions, immunosuppression, and transfusion-related acute lung injury, widespread adoption of ICS during oncologic surgery remains controversial. Concerns persist regarding the reinfusion of malignant cells and the theoretical risk of metastatic disease.

Over the past decades, many studies on ICS in diverse oncologic procedures have been performed and have subsequently been synthesized in several systematic reviews. Furthermore, the use of ICS during oncologic surgery has been endorsed by several clinical guidelines. Here, we provide a comprehensive and up-to-date overview of current evidence regarding the use of ICS during oncologic surgery. Evidence was retrieved by a PubMed search using the terms ‘intra-operative cell salvage,’ ‘autologous transfusion’, ‘oncologic surgery’, and we complemented this with manual screening of reference lists, guidelines, and previously published systematic reviews.

In this review, we discuss historical developments, underlying principles, available evidence from in vitro and clinical studies, existing clinical guidelines, and conclude with knowledge gaps and future directions.

## 2. History of ICS

The concept of reinfusing a patient’s own blood dates back over two centuries. A British obstetrician named James Blundell, performed and documented extensive transfusion experiments with animals and humans in the early 19th century and developed instruments to collect and reinfuse blood [1,2]. He is sometimes credited with the first reinfusion of a patient’s own lost blood to treat post-partum hemorrhage [3,4], although this is presumably erroneous [5]. In the decades to follow, various physicians, including William Halsted in 1883 and John Duncan in 1886, performed the first autologous transfusions in and outside of the operating theater [6,7]. The first improvised device for cell salvage and autotransfusion was developed by Arnold Griswold in 1943, using glass bottles to collect suctioned blood and a cheesecloth to filter salvaged blood [8]. Further developments in technology followed in the 1960s, when centrifugal processing was introduced to separate red cells from other blood components. Jack Latham presented the concept of a centrifugal bowl—known as the “Latham bowl”—which is still in use in many devices today [9,10]. Commercial production of modern cell salvage machines started in the 1970s. These devices enabled washing, concentration, and reinfusion of autologous red cells. In 1974, the Bentley Autotransfusion System, which was the first commercially available device, was withdrawn from the market due to concerns about air embolism and coagulation disorders [11]. Since then, no major complications have been reported with newer systems.

The first reported use of ICS in oncologic surgery was in 1975. A 52-year-old male patient undergoing a lobectomy via right thoracotomy for lung carcinoma, had 1000 mL of blood collected via an autotransfusion system [12]. Although the salvaged blood was not reinfused, cytological analysis revealed tumor cells in both filtered and unfiltered samples. These cells shared morphological characteristics with the resected tumor tissue, indicating that malignant cells could pass through the ICS system. Based on these findings, the authors advised against the use of ICS in procedures involving known or suspected malignancy [12]. This case report contributed to the Council on Scientific Affairs’ recommendation to avoid ICS in surgeries where blood may be contaminated with tumor cells [11]. As a result, ICS was largely not used for oncologic surgery in the following decades due to fear of metastases.

## 3. Principles of ICS

ICS is a three-stage process designed to collect, centrifuge, and reinfuse autologous red blood cells (RBCs) lost during surgery. The process begins with the aspiration of shed blood from the operative field using specialized low-pressure suction systems. This blood is immediately anticoagulated—typically with heparinized saline or citrate-based solutions—and passed through a filter to remove debris. Once collected in a reservoir, the anticoagulated blood undergoes separation in a centrifuge bowl or disk system. Here, red cells are isolated from lighter components such as plasma, platelets, and cellular parts, which are discarded into a waste bag. The remaining RBCs are then washed with isotonic saline to remove any residual anticoagulants, free hemoglobin, and inflammatory mediators. The final product is a concentrated suspension of autologous RBCs in saline, typically with a hematocrit between 50–80% [4]. Compared to ABT, salvaged RBCs maintain better deformability, oxygen delivery capacity, and have higher concentrations of 2,3-diphosphoglycerate [4,13,14].

## 4. Leukocyte Depletion Filters

After processing, the salvaged RBCs are often passed through leukocyte depletion filters (LDFs), before being reinfused to the patient. Originally developed in the 1990s, current fourth-generation models are in use, combining filtration and electrostatic adsorption to achieve high levels of cellular removal [15,16]. Structurally, they consist of densely packed microfiber layers with pore sizes ranging from 3 to 8 micrometers [17]—compared to 40 micrometers of standard blood filters—enabling the removal of leukocytes and other cells. Although highly effective, earlier LDFs had practical limitations due to their low flow rates. Modern fourth-generation filters, however, allow for transfusion rates of approximately 50 mL per minute. To ensure optimal performance, they should be replaced after processing around 450 mL of blood [14,18].

## 5. Circulating Tumor Cells

In patients undergoing oncologic surgery, the presence of circulating tumor cells (CTCs) in the bloodstream already prior to surgery is reported in various malignancies, including breast, colorectal, hepatocellular, pancreatic, prostate and lung cancers [19,20,21,22,23,24,25,26,27]. Elevated baseline CTC counts in non-metastatic, early-stage cancer predict increased recurrence rates, and shorter disease-free and overall survival [26,27,28,29,30,31]. Furthermore, the number of CTCs is of prognostic relevance. Studies in non-metastatic prostate, colorectal and breast cancer report that the number of CTCs are independently associated with worse progression-free and overall survival [19,32,33,34,35]. These findings support the concept that hematogenous dissemination may occur early in tumor development, before radiologic or clinical signs of metastasis are apparent.

While the detection of CTCs confirms tumor cell dissemination, it does not necessarily predict metastatic burden. The metastatic cascade is highly complex and inefficient, with fewer than 0.01% of CTCs, ultimately leading to established metastases [36]. Most are cleared by host defenses or lack the molecular traits needed for extravasation and colonization. However, certain CTCs—particularly those with stemness and mesenchymal transition traits—may have enhanced metastatic potential [37].

Surgical manipulation may increase the number of tumor cells released into circulation. Intra- and postoperative rises in CTC counts have been observed across multiple cancer types, including colorectal and lung cancer [38,39,40,41]. These elevations are believed to result from mechanical disruption of the tumor during resection. However, the clinical relevance of these surgery-induced rises in CTCs remains unclear. Some studies have associated postoperative CTC increases with shorter recurrence-free survival [41], others have found no correlation with long-term outcomes [42].

## 6. ABT and Outcome

During oncologic surgery, the treatment of perioperative anemia below the transfusion threshold, primarily consisted of ABT. Several meta-analyses and retrospective studies, however, demonstrate that ABT is associated with worse overall survival, increased recurrence rates, and elevated cancer-specific mortality. For instance, meta-analyses in hepatocellular carcinoma [43], gastric and colorectal cancer [44,45], and urologic malignancy cohorts [46,47,48] demonstrated significantly increased risks of both recurrence and mortality among transfused patients. Although some heterogeneity exists between studies and cancer types, the overarching trend consistently associates ABT with inferior long-term outcomes. Biological mechanisms that may underlie these associations include transfusion-related immunomodulation, perioperative immunosuppression, and increased systemic inflammation [49]. Furthermore, both anemia and ABT are associated with tumor progression, underlining the potential for ICS [50].

## 7. ICS

The use of ICS in perioperative care—primarily non-oncologic surgery—has extensively been studied over the last decades in randomized controlled trials (RCTs). The most recent Cochrane review on ICS across a variety of surgical procedures concluded, based on 82 RCTs involving 12,520 patients, that ICS is associated with an overall reduced risk of ABT (RR 0.65; 95% CI 0.59–0.72; *p* < 0.00001) [51]. However, the authors noted that the certainty of this conclusion is limited due to the generally low quality of the included studies. ICS appears to have the most benefit in reducing ABT requirements among patients undergoing cardiovascular or spine surgery, while evidence remains uncertain for other surgical categories [51].

Importantly, only two RCTs in this review were studies on patients with malignancies [52,53]. These studies had a very small sample size, including a total of 79 patients. One was a feasibility study in patients with ovarian cancer, for which only an abstract has been published and final results are still pending [53]. The other was a study conducted in 1997, randomizing 24 patients undergoing prostate cancer surgery to ICS or standard care [52]. Although fewer units of allogeneic blood were transfused in the ICS group, the study primarily assessed laboratory parameters without reporting any long-term clinical follow-up. Therefore, current clinical evidence regarding the safety and efficacy of ICS in oncologic surgery can only be derived from in vitro and observational studies.

## 8. In Vitro Studies

Several studies have demonstrated that the mechanical stressors involved in ICS processing significantly compromise the structural integrity and viability of tumor cells. Following processing through ICS devices, tumor cells often appear as fragmented cytoplasmic debris, lacking intact nuclei or proliferative capability [54]. For instance, Karczewski et al. reported that approximately 62% of tumor cells sustained lethal injury during processing, while the remaining cells exhibited marked morphological alteration [55]. More recent studies have replicated these findings, showing that blood samples processed failed to generate tumor cell clusters in culture—unlike samples drawn directly from the venous system or the operative field prior to filtration [56]. These findings support the notion that, although occasional circulating tumor cells may be present in salvaged blood, the trauma induced during ICS is sufficient to render them nonviable and unlikely to initiate metastatic spread.

Many in vitro studies—without reinfusion of the salvaged blood to the patient—have shown that LDFs can effectively remove tumor cells from intraoperative salvaged blood. Early work by Perseghin et al. demonstrated that LDFs achieved a complete removal of neoplastic cells after filtration in lung cancer patients [57]. Similar results were seen in gynecologic oncology: Catling et al. found that while 68% of reservoir samples contained malignant cells, no viable tumor cells were detectable after filtration with an LDF [54]. Similar results were obtained in hepatocellular and pancreatic carcinoma [58], breast [59], urologic [60], and gynecologic cancer [61], metastatic spine tumor surgery [56], and a combination of various malignant cell lines [62].

## 9. Tumor Cells Detectable After LDFs

Although the majority of in vitro studies have reported no presence of malignant cells after processing through an LDF, several studies have shown that complete elimination cannot always be achieved, though these occurred under laboratory conditions that used cell loads far higher than those encountered in clinical practice. For instance, Frühauf et al. performed a series of in vitro experiments using various gastrointestinal tumor cells added to either saline or whole blood, and reported a 99.96% median depletion rate after filtration. However, in 1 out of 14 experiments in which tumor cell lines were admixed with whole blood, complete depletion was not achieved. This involved the sample with the highest number of tumor cells, suggesting that some cells may pass through depending on the initial load and filter saturation [63]. Similarly, Gwak et al. tested the efficacy of an LDF using hepatocellular carcinoma cell lines. While filtration significantly reduced detectable tumor cells, they observed that under high cell loads (2 × 10^5^–2 × 10^7^ cells per 200 mL), the LDF was unable to completely eliminate all malignant cells [64]. They hypothesized that the absolute number of tumor cells passing through the filter was likely very small, as they had used a highly sensitive detection technique and reamplification was required to confirm tumor cell presence. In a more recent study involving 15 patients undergoing liver transplantation, viable tumor cells were identified in the filtered blood of two patients—those with the highest and second-highest tumor loads. The authors speculated that these high tumor loads may have exceeded the filtration capacity of the LDF [65].

In another study involving prostate cancer patients, tumor cells were present in 2 out of 15 samples after filtration [66]. Only after subsequent irradiation tumor cells were no longer detectable, suggesting that filtration alone may not guarantee complete tumor cell removal. Similarly, in a study of eight patients undergoing metastatic spine tumor surgery, one patient still had detectable tumor cells after filtration [67]. This patient had a poorly differentiated squamous cell lung carcinoma with thoracic vertebral metastases, and also the shortest survival of just two months, possibly indicating a high preoperative tumor load.

## 10. Irradiation

In addition to filtration, irradiation has been proposed as an adjunct to eliminate malignant cells from salvaged blood. Several in vitro studies have demonstrated that irradiation, typically at doses ranging from 25 to 100 Gy, can render tumor cells mitotically inactive while preserving erythrocyte morphology and function. Early experimental work by Hansen et al. showed complete tumor cell inactivation following gamma irradiation with 50 Gy in various cell lines from solid tumors [68]. These results were subsequently confirmed by two other studies [69,70]. One used hepatocellular and gastro-intestinal tumor cell lines added to salvaged blood from healthy volunteers, which was irradiated with 30, 50, or 100 Gy. They observed a dose-dependent inhibition of tumor cell viability, proliferation, and tumorigenicity, and no xenograft tumors were observed in mice injected with irradiated cells. Erythrocyte morphology, free hemoglobin levels, and 2,3-diphosphoglycerate concentrations remained unaffected [69]. In a similar study, salvaged blood mixed with hepatocellular carcinoma cells was irradiated with 30 and 50 Gy, with both doses completely suppressing tumor cell viability, DNA synthesis, and tumorigenicity in xenograft models, while preserving erythrocyte function [70].

In comparison with filtration through an LDF, conflicting results have been reported. Poli et al. used a prostate cancer-specific methylation marker to detect tumor DNA in salvaged blood and found that irradiation eliminated all detectable tumor signal, whereas filtration through an LDF did not [66]. In contrast, a study testing both 25 Gy and 100 Gy gamma doses reported that irradiation did not reduce tumor-specific mRNA in gastrointestinal cancer cells, whereas filtration with an LDF successfully removed all detectable mRNA from the same spiked blood samples, suggesting filtration may provide more consistent removal [71].

Clinical data on irradiation are sparse, but a retrospective study by Weller et al. evaluated 51 patients undergoing liver transplantation, comparing patients who received irradiated salvaged blood to a historical control group that received unfiltered salvaged blood. The patients who received irradiated blood showed no significant difference in recurrence-free survival compared to controls, although the small sample size limits definitive conclusions [72]. Despite theoretical benefits and promising experimental data, irradiation is rarely used in routine practice. This is largely due to logistical challenges, such as the need for dedicated irradiators in or close to the operating room, potential transport delays, radiation safety regulations, and additional training for personnel.

## 11. Observational Studies

Many observational studies on ICS with or without LDF and irradiation have been performed. Their findings have been aggregated in eleven systematic reviews [73,74,75,76,77,78,79,80,81,82,83], of which the main results are summarized in Table 1. Most reviews concentrated on hepatocellular carcinoma or urologic malignancies, although several also included gynecologic and gastrointestinal cancers. Collectively, these reviews included large overall cohorts, yet the underlying studies differed markedly in sample size. Follow-up periods were likewise heterogeneous, ranging from a few months to more than ten years. Only a minority of included studies reported using an LDF, and just one implemented irradiation in clinical practice [72]. Study quality or risk of bias was formally assessed in nine of the eleven reviews—both were predominantly rated as moderate, and the overall certainty of the evidence was deemed low.

Interestingly, four reviews report reduced recurrence rates in hepatocellular carcinoma [73,77,79,81]. Unlike the predominantly urologic tumors in the other reviews, hepatocellular carcinoma has a greater metastatic potential. In studies involving urologic cancers, no differences in outcomes were observed with the use of ICS. The reduced recurrence rates in hepatocellular carcinoma may be resulting from a decreased need for ABT during surgery—these tumors are notorious for high perioperative transfusion requirements, and greater ABT exposure is linked to poorer outcomes [43].

None of these reviews included RCTs, and the vast majority are based on retrospective observational studies, which are inherently prone to bias and were often limited by small cohort sizes. Furthermore, not all retrospective studies applied statistical methods such as propensity score matching to account for selection bias. Of note, some reviews, primarily those on hepatocellular carcinoma, have included the same cohort studies, thereby potentially replicating each other’s conclusions. Despite these methodological shortcomings, the conclusions across all reviews are consistent: ICS is not associated with increased risk of cancer recurrence or mortality, and may offer clinical advantages by reducing the need for ABT. All systematic reviews thus support the use of ICS during oncologic surgery, particularly when combined with filtration through an LDF.

## 12. Guidelines

Based on in vitro and clinical evidence, the use of ICS during oncologic surgery has been supported by national and international guidelines. The UK Association of Anaesthetists recommends ICS when it is expected to reduce ABT requirements or prevent severe postoperative anemia, particularly when estimated blood loss exceeds 500 mL. In cases involving malignancy or infection, ICS should only be performed with explicit patient consent, and the use of LDFs should be considered [84].

The National Institute for Health and Care Excellence limits its recommendations to ICS use to radical prostatectomy or cystectomy. They state that ICS may be used in these procedures, and that patients must receive clear, written information comparing the risks and benefits of ICS versus ABT [85].

Australia’s National Blood Authority recommends ICS in a wide range of elective and emergency surgeries where blood loss is anticipated to exceed 1000 mL or 20% of blood volume. In oncologic surgery, the use of ICS is not contraindicated, provided that LDFs are used and informed consent is obtained preoperatively [86].

Similarly, the European Society of Anaesthesiology and Intensive Care states that ICS is not contraindicated in oncologic surgery, as long as aspiration near the tumor site is avoided and an LDF is used. They recommend considering ICS in high-bleeding-risk oncologic procedures, including gynecological and hepatic surgery, while acknowledging the need for further prospective data [87]. The Italian Society of Transfusion Medicine and Immunohematology further recommends that salvaged blood be both filtered and irradiated (at 25 Gy) before reinfusion, particularly in cases involving high tumor burden [88].

Finally, the North American Society of Thoracic Surgeons permits ICS during cancer surgery involving cardiopulmonary bypass. Although based primarily on data from non-oncologic cardiothoracic procedures, these guidelines acknowledge improved outcomes in patients receiving ICS and support its use even in the absence of cancer-specific clinical data [89].

## 13. Conclusions and Future Perspectives

ICS has been available for several decades and its use is associated with reduced need for ABT. Although the available evidence regarding the use of ICS in oncologic surgery is largely based on moderate quality observational studies, consistent findings across numerous studies support its safety. CTCs are frequently present in patients with non-metastatic disease and increase following surgical manipulation. Moreover, tumor cells are present in blood suctioned from the tumor site, but are morphologically altered during processing through a cell salvage apparatus. Filtration through LDFs is highly effective in removing tumor cells, although complete elimination cannot always be guaranteed. Further irradiation may be considered, albeit this entails some logistical issues. Nonetheless, the theoretical risk of metastasis from ICS appears small. No clinical cases of metastasis directly attributable to ICS have ever been reported, although this would be difficult to prove. Overall, ICS appears to be a safe and potentially beneficial strategy for perioperative blood management in oncologic surgery, reducing exposure to ABT and their associated risks. This view is shared by national and international guidelines, all of which support the use of ICS in oncologic surgery. However, the certainty of these conclusions remains limited by the absence of high-quality evidence, preferably via randomized controlled trials. A definitive prospective, preferably multi-center, study would require a large sample size (estimated at >1000 patients [90]) and long-term follow-up, making such a trial logistically and financially challenging. Embedding such a trial within an international surgical quality-improvement network potentially could make it feasible, yet no trial has been registered yet, to our knowledge.

Despite these limitations and awaiting a definitive RCT, the current body of evidence supports the safety and utility of ICS in oncologic surgery. Wider clinical adoption, therefore, appears justified and should be considered where feasible.

## Figures and Tables

**Table 1:** Summary of data of the available systematic reviews on intraoperative cell salvage.

First Author, Year	Number of Included Studies	Total Number of Patients Included	Study Sample Size Range	Cancer Type	Study Design	Number of Studies Reported Use of an LDF	Reported Follow-Up Range	Risk of Bias/Quality of Evidence	Main Results
Waters, 2012 [73]	11	2390 (769 received ICS)	47–1038	Hepatic, cervical, prostate, gastrointestinal	Prospective observational (n = 4) Retrospective (n = 7)	NS	1–120 months	NS	*Studies analyzing cancer recurrence (n = 10):* Reduced recurrence rates in patients receiving ICS compared to controls (OR 0.65; 95% CI 0.43–0.98; *p* = 0.04). *Prostate cancer studies (n = 5):* No difference in recurrence rates in patients receiving ICS compared to controls (OR 0.54; 95% CI 0.27–1.07; *p* = 0.08).
Kumar, 2014 [74]	30	6888 (1860 received ICS with reinfusion and 133 without reinfusion)	9–1862	Gynecological, hepatobiliary, gastrointestinal, urologic, lung	Prospective observational (n = 17) Retrospective (n = 11) In vitro (n = 2)	NS	0–61 months	NS	*Gynecological cancer studies (n = 4)* Reduced ABT requirements in patients receiving ICS compared to controls, without effect on metastasis or recurrence. *Hepatobiliary cancer studies (n = 7):* Reduced ABT requirements in patients receiving ICS compared to controls, without effect on recurrence or survival. *Gastrointestinal cancer studies (n = 1):* Reduced ABT requirements in patients receiving ICS compared to controls, without effect on metastasis or recurrence. *Urological cancer studies (n = 15):* Reduced ABT requirements in patients receiving ICS compared to controls, without increasing recurrence, metastasis, or mortality. *Lung cancer studies (n = 1):* LDFs effectively removed malignant cells from salvaged blood. *In vitro studies (n = 2):* LDFs effectively removed malignant cells from blood mixtures while standard blood filters did not.
Kinnear, 2018 [75]	14	4536 (1223 received ICS)	27–1862	Urologic	Prospective observational (n = 2) Retrospective (n = 12)	6	0–65 months	NOS: - 5 stars (n = 1) - 6 stars (n = 3) - 7 stars (n = 1) - 8 stars (n = 6) - 9 stars (n = 3) Conclusion by the authors: 10 studies rated as low risk of bias, 4 studies as moderate risk of bias.	*Prostatectomy studies (n = 4):* Reduced ABT requirements in patients receiving ICS compared to controls (OR 0.34; 95% CI 0.15–0.76; *p* = 0.01). *Studies analyzing oncological outcomes (n = 10):* No difference (n = 8 studies) or improved outcomes (n = 2 studies) in patients receiving ICS compared to controls. *Cystectomy and partial nephrectomy studies (n = 2):* No difference in complication rates (39.5% vs. 40.5% after cystectomy and 21% vs. 17% after partial nephrectomy) in patients receiving ICS compared to controls. *Studies analyzing costs (n = 2):* ICS was cheaper than no blood conservation technique (GBP 320 vs. GBP 675 and GBP 163 vs. GBP 673 per patient).
Guo, 2018 [76]	8	1755 (662 received ICS)	47–397	Hepatic	Retrospective (n = 8)	1	0.5–134 months	NOS: - 6 stars (n = 1) - 7 stars (n = 3) - 8 stars (n = 4) Conclusion by the authors: Studies rated as moderate to high quality.	*Studies analyzing recurrence rates (n = 7):* No difference in recurrence rates in patients receiving ICS compared to controls (RR 0.85; 95% CI 0.71–1.02; *p* = 0.69). *Studies analyzing recurrence free survival (n = 5):* Improved recurrence-free survival in patients receiving ICS compared to controls (RR 1.18; 95% CI 1.03–1.36; *p* = 0.15). *Studies analyzing mortality (n = 5):* No difference in mortality in patients receiving ICS compared to controls (HR 0.80; 95% CI 0.58–1.11; *p* = 0.84).
Wu, 2019 [77]	9	4354 (1346 received ICS)	71–1862	Urologic, cervical, hepatic	Prospective observational (n = 1) Retrospective (n = 8)	NS	NS	NOS: - 5 stars (n = 3) - 6 stars (n = 3) - 7 stars (n = 3) Conclusion by the authors: 6 studies rated as moderate quality, 3 studies as high quality.	*Studies analyzing overall survival (n = 4):* No difference in five-year overall survival in patients receiving ICS compared to controls (OR 1.12; 95% CI 0.80–1.58; *p* = 0.51). *Studies analyzing disease-free survival (n = 3):* No difference in five-year disease-free survival in patients receiving ICS compared to controls (OR 1.08; 95% CI 0.57–1.67; *p* = 0.53). *Studies analyzing recurrence rates (n = 6):* No difference in five-year recurrence rates in patients receiving ICS compared to controls (OR 0.86; 95% CI 0.71–1.05; *p* = 0.15). *Liver transplantation studies (n = 4):* No difference in five-year overall survival in patients receiving ICS compared to controls (2 studies; OR 0.97; 95% CI 0.57–1.67; *p* = 0.92). Reduced five-year recurrence rates in patients receiving ICS compared to controls (4 studies; OR 0.65; 95% CI 0.46–0.92; *p* = 0.02).
Aijtink, 2022 [78]	9	1997 (1200 received ICS)	52–397	Hepatic	Retrospective (n = 9)	5	18–78 months	ROBINS-I: - Low risk of bias (n = 2) - Moderate risk of bias (n = 5) - Serious risk of bias (n = 2) Conclusion by the authors: Studies predominantly rated as moderate quality.	*Studies analyzing disease-free survival (n = 4):* No difference in disease-free survival in patients receiving ICS compared to controls (HR 0.90; 95% CI 0.66–1.24; *p* = 0.53). *Studies analyzing recurrence rates (n = 5):* No difference in recurrence rates in patients receiving ICS compared to controls (HR 0.83; 95% CI 0.57–1.23; *p* = 0.36). *Studies analyzing overall survival (n = 4):* No difference in overall survival in patients receiving ICS compared to controls compared to controls (HR 1.07; 95% CI 0.70–1.62, *p* = 0.75).
Frietsch, 2022 [79]	34	8503 (3161 received ICS)	16–395	Hepatic, renal, prostate, bladder, cervical, colorectal, pancreatic, liver metastases, metastatic spine tumors, gastrointestinal, growth or lung metastases from kidney cancer or sarcoma	Prospective observational (n = 12) Retrospective (n = 22)	11	9–96 months	GRADE: - Very low quality (n = 1) - Very low to low quality (n = 1) - Low quality (n = 28) - Low to moderate quality (n = 3) - NS (n = 1) Conclusion by the authors: Studies predominantly rated as low quality and risk of bias considered high, since all included studies were observational.	*Studies analyzing recurrence rates (n = 25):* Reduced recurrence rates in patients receiving ICS compared to controls (OR 0.76; 95% CI 0.64–0.90). *Studies analyzing mortality (n = 20):* No difference in mortality in patients receiving ICS compared to controls (OR 0.95; 95% CI 0.71–1.27). *Studies analyzing LOS (n = 10):* No difference in LOS in patients receiving ICS compared to controls (mean difference 0.07 days; 95% CI −0.63–0.48).
Murtha-Lemekhova, 2022 [80]	14	1314 (803 received ICS)	47–319	Hepatic	Prospective observational (n = 1) Retrospective (n = 9)	1	NS	ROBINS-I: - Low risk of bias (n = 11) - Moderate risk of bias (n = 3) Conclusion by the authors: Studies predominantly rated as moderate risk of bias. Certainty of evidence, according to GRADE, rated as very low.	*Studies analyzing overall survival (n = 6):* No difference in overall survival in patients receiving ICS compared to controls (HR 1.13; 95% CI 0.89–1.42; *p* = 0.31). *Studies analyzing disease-free survival (n = 8):* No difference in disease-free survival in patients receiving ICS compared to controls (HR 0.97; 95% CI 0.76–1.24; *p* = 0.83). *Studies analyzing recurrence rates (n = 6):* No difference in recurrence rates in patients receiving ICS compared to controls (OR 0.71; 95% CI 0.41–1.23; *p* = 0.22).
Wang, 2022 [81]	12	2253 (1374 received ICS)	23–397	Hepatic	Retrospective (n = 12)	6	NS	NOS: - 6 stars (n = 1) - 7 stars (n = 5) - 8 stars (n = 5) - 9 stars (n = 1) Conclusion by the authors: 1 study rated as moderate quality, 11 studies as high quality.	*Studies analyzing recurrence rates:* Reduced five-year recurrence rates in patients receiving ICS compared to controls (9 studies; OR 0.75; 95% CI 0.59–0.95; *p* = 0.02). Reduced 7-year recurrence rates in patients receiving ICS compared to controls (5 studies; OR 0.65; 95% CI 0.44–0.95; *p* = 0.03). Reduced five-year recurrence rates in patients receiving ICS with an LDF compared to controls (6 studies; OR 0.73; 95% CI 0.55–0.96; *p* = 0.03). *Studies analyzing overall survival (n = 7):* No difference in five-year overall survival in patients receiving ICS compared to controls (OR 1.04; 95% CI 0.76–1.40; *p* = 0.82). *Studies analyzing disease-free survival (n = 2):* No difference in five-year disease-free survival in patients receiving ICS compared to controls (OR 0.88; 95% CI 0.60–1.28; *p* = 0.50).
Rajendran, 2023 [82]	21	3433 (1445 received ICS)	41–670	Hepatic, colorectal metastases	Retrospective (n = 21)	5	12–120 months	MINORS: - 14 (n = 1) - 15 (n = 2) - 16 (n = 2) - 17 (n = 8) - 18 (n = 4) - 19 (n = 3) - 20 (n = 1) Conclusion by the authors: 16 studies rated as low risk of bias, 5 studies rated as high risk of bias. Certainty of evidence, according to GRADE, rated as very low to low.	*Studies analyzing ABT requirements (n = 6): * No difference in ABT requirements (number of units) in patients receiving ICS compared to controls (mean difference −1.28 units; 95% CI −3.26–0.70; *p* = 0.20). *Studies analyzing overall survival: * No difference in overall survival in liver transplant patients receiving ICS compared to controls (3 studies; HR 1.10; 95% CI 0.67–1.79; *p* = 0.71). No difference in overall survival in liver resection patients receiving ICS compared to controls (2 studies; HR 0.69; 95% CI 0.45–1.05; *p* = 0.08). *Studies analyzing disease-free survival: * No difference in disease-free survival in liver transplant patients receiving ICS compared to controls (5 studies; HR 0.93; 95% CI 0.57–1.49; *p* = 0.75). No difference in disease-free survival in liver resection patients receiving ICS compared to controls (2 studies; HR 0.89; 95% CI 0.43–1.85; *p* = 0.75).
Hinojosa-Gonzalez, 2024 [83]	12	1704 (969 received ICS)	23–319	Hepatic	Retrospective (n = 12)	5	NS	NOS: - 6 stars (n = 2) - 7 stars (n = 8) - 8 stars (n = 2) Conclusion by the authors: 2 studies rated as moderate quality, 10 studies as high quality.	*Studies analyzing ABT requirements (n = 7): * No difference in ABT requirements (number of units) in patients receiving ICS compared to controls (mean difference −0.56 units; 95% CI −1.89–0.78; *p* = 0.41). *Studies analyzing recurrence rates: * No difference in recurrence rates in patients receiving ICS without an LDF compared to controls (6 studies; HR 0.82; 95% CI 0.59–1.15; *p* = 0.25). No difference in recurrence rates in patients receiving ICS with an LDF compared to controls (5 studies; HR 0.81; 95% CI 0.59–1.12; *p* = 0.20). *Studies analyzing 1-year recurrence-free survival (n = 11):* No difference in 1-year recurrence-free survival in patients receiving ICS compared to controls (HR 0.89; 95% CI 0.63–1.25; *p* = 0.50). *Studies analyzing overall survival (n = 5):* No difference in overall survival in patients receiving ICS compared to controls (HR 0.86; 95% CI 0.66–1.13; *p* = 0.29).

ABT: allogeneic blood transfusion; CI: confidence interval; GRADE: Grading of Recommendations, Assessment, Development, and Evaluations; HR: hazard ratio; ICS: intraoperative cell salvage; LDF: leukocyte depletion filter; LOS: length of stay; MINORS: Methodological Index for Non-Randomized Studies; NOS: Newcastle-Ottawa Scale; NS: not specified; OR: odds ratio; ROBINS-I: Risk Of Bias in Non-Randomised Studies - of Interventions; RR: relative risk.
